# Effects of Fixation and Drying on the Physicochemical Quality and Aroma Profiles of Vine Tea (*Nekemias grossedentata*): Identification of Critical Processing Steps for Flavor Formation

**DOI:** 10.3390/foods15173067

**Published:** 2026-08-29

**Authors:** Fei Ye, Kui Chen, Anhui Gui, Yayan Yu, Chaoyang Zhang, Panpan Liu, Xueping Wang, Lin Feng, Jin Teng, Jinjin Xue, Pengcheng Zheng, Shiwei Gao

**Affiliations:** 1 Fruit and Tea Research Institute, Hubei Academy of Agricultural Sciences, Wuhan 430064, China; yf421@163.com (F.Y.);; 2 Laifeng County Agricultural and Rural Bureau, Laifeng 445700, China; 3 Key Laboratory of Tea Science, Ministry of Education, Hunan Agricultural University, Changsha 410128, China; 4 Enshi Tujia and Miao Autonomous Prefecture Academy of Agricultural Sciences, Enshi 445000, China

**Keywords:** vine tea, fixation, drying, physicochemical quality, flavor formation, volatile organic compounds, relative odor activity value, HS-SPME-GC-MS-O

## Abstract

The quality and flavor of vine tea are largely determined by its processing stages, which markedly influence its physical attributes and volatile organic compounds. Elucidating the dynamic changes in physicochemical and aromatic properties throughout processing is, therefore, essential for guiding optimized processing techniques and developing high-quality vine tea products. However, the specific effects of individual processing stages on quality attributes remain poorly understood. In this study, we combined assessments of color and physical properties with untargeted metabolomics, headspace solid-phase microextraction coupled with gas chromatography–mass spectrometry (HS-SPME-GC-MS), relative odor activity value (ROAV), and gas chromatography–olfactometry (GC-O) to identify key compounds contributing to vine tea quality. Principal component analysis (PCA) and partial least squares discriminant analysis (PLS-DA) were further applied to identify characteristic metabolites associated with aroma and flavor differentiation. A total of 280 volatile organic compounds were identified, among which 14 key VOCs (ROAV ≥ 1, aroma intensity ≥ 0.5) exhibited significant dynamic variation across processing stages. Notably, compounds such as β-ionone, β-myrcene, nonanal, and hexanal displayed higher ROAVs and strong aroma intensities (AI ≥ 1.0), indicating their substantial contribution to overall aroma. Furthermore, the metabolic transformation pathways—primarily including fatty acid degradation and carotenoid cleavage—and the content changes of key aroma-active compounds were inferred across different processing stages. Based on the differential accumulation patterns of the identified volatile markers, the possible involvement of fatty acid degradation and carotenoid cleavage pathways was inferred. Drying and fixation emerged as critical steps for vine tea aroma development, while the non-enzymatic degradation of fatty acids was potentially associated with the formation of its aroma characteristics. This study provides insights that may inform future efforts toward processing optimization and quality improvement of vine tea.

## 1. Introduction

Vine tea (*Nekemias grossedentata*), a non-Camellia herbal tea traditionally known as Mei tea or Duanwu tea, is widely distributed across central and southern China and has been consumed for centuries as a health-promoting beverage [1]. It is distinguished by its exceptionally high content of dihydromyricetin (DHM, >18% dry weight), which exhibits antioxidant, anti-inflammatory, and hepatoprotective activities [2,3,4], attracting growing interest in the functional food industry [5]. However, compared to Camellia teas, vine tea remains understudied regarding its processing-induced aroma formation.

Vine tea processing generally follows procedures similar to green or yellow teas—fixation, rolling, stacking, and drying [6]—each of which profoundly influences the final sensory properties. Aroma is a decisive sensory attribute that directly determines consumer acceptance [7], generated through biochemical and thermal reactions (lipoxygenase pathway LOX, Maillard reaction, carotenoid degradation, and glycosidic hydrolysis) closely linked to specific processing conditions. Extensive research on Camellia sinensis has established that fixation preserves green color and promotes aldehydes and pyrazines [8,9,10]; the yellow tea stacking step reduces astringency and increases sweetish aroma [11]; and black tea fermentation generates floral volatiles like *β*-ionone and linalool [8]. Yet no such systematic investigation exists for vine tea.

To date, only limited studies have examined vine tea volatiles, identifying aldehydes, ketones, esters, and alcohols as major components [12,13,14], but none have tracked volatile evolution across the entire processing chain or integrated sensory validation. Critical gaps remain: (i) the dynamic changes in volatiles and non-volatiles across processing stages are unknown; (ii) the key steps and mechanisms responsible for aroma formation are unclear; and (iii) the correlations between chemical changes and sensory quality have not been established. Recent work has advanced understanding of vine tea volatiles [13,14], but none has employed GC-O as direct sensory validation or explicitly linked processing steps to aroma formation pathways.

To address these gaps, the present study introduces an integrated analytical framework combining colorimetry, physicochemical profiling, HS-SPME-GC-MS, GC-O, and ROAV analysis. This approach enables: (i) temporal mapping of volatile and non-volatile compounds across the processing chain; (ii) identification of processing stages critical for aroma formation; and (iii) characterization of dominant aroma-active compounds and their sensory contributions. By constructing a processing pathway-oriented framework, our findings provide a reference for understanding aroma formation during processing and may assist in the rational design of future quality improvement strategies.

## 2. Materials and Methods

### 2.1. Experimental Materials and Chemical Standards

#### 2.1.1. Vine Tea Samples and Process Characterization

**Figure 1:**
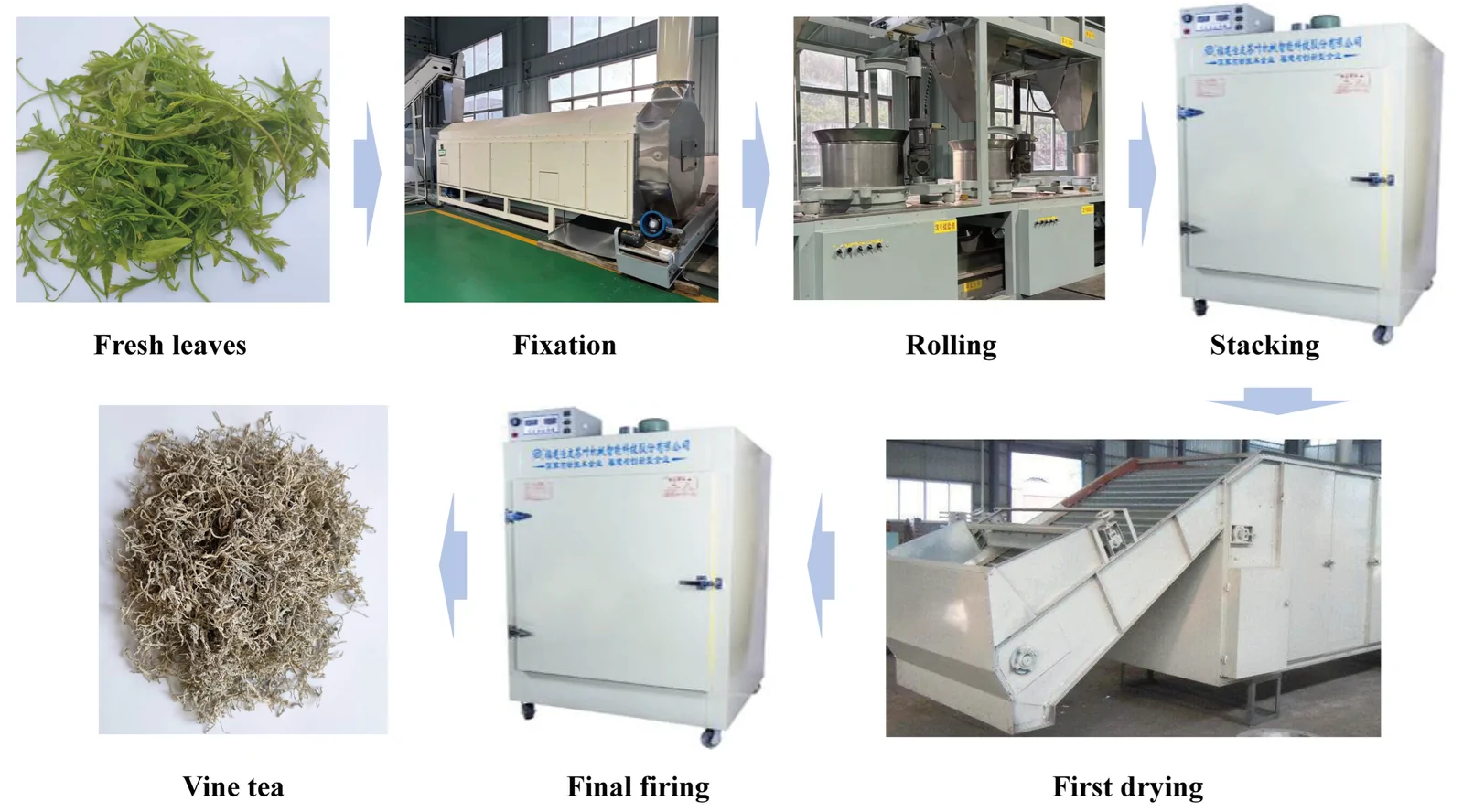
Flowchart of vine tea processing.

Fresh tender shoots (consisting of the young leaves and a short tender stem) of *Nekemias grossedentata* were harvested on 10 May 2024. Approximately 200 kg of vine tea leaves (8.0–10.0 cm long) were harvested in Laifeng County, Hubei, China. The average moisture content of fresh leaves was 78.5% ± 0.5% (w.b.). Leaves were free from visible disease or mechanical damage and were transported to the processing facility within 2 h; they were processed locally following conventional fixation, rolling, stacking, and drying procedures. The flowchart of vine tea processing is detailed below (Figure 1).

(1) Fixation: Leaves were fixed in a hot-air roller (6CSF-100, Zhejiang Feng Kai Co., Ltd., Hangzhou, China) at air temperatures of 250–260 °C, a roller speed of 24 rpm, and for a duration of 150–155 s. After fixation, the leaf moisture content reached ~54–55%; fixed leaves were then cooled/softened in a cooling device (YJY-20, Ningbo Yaojiangyuan Machinery Co., Ltd., Ningbo, China).

(2) Rolling: Fixed leaves were rolled in a rolling machine (6CR-55, Zhejiang Shang Yang Co., Ltd., Quzhou, China) for 2 min at 28 rpm.

(3) Stacking: Rolled leaves were sealed in plastic bags and placed in a box hot-air drying device (JY-6CHZ-7B, Jiayou Machinery Co., Ltd., Quanzhou, China) at 30 °C for 12 h, until a white frost developed on the leaves. It is hypothesized that moisture loss during stacking might promote DHM deposition on the leaf surface, and the vine tea becomes white, although this proposed mechanism requires further experimental substantiation.

(4) Drying: including first drying and final firing, leaves were first dried in a chain-plate dryer (6CHB-10, Zhejiang Shang Yang Co., Ltd., Quzhou, China) at 100 °C for 15 min to reduce moisture to ~30–33%, followed by 30 min of outdoor cooling. The final firing step was performed in the box hot-air dryer (JY-6CHZ-7B) at 90 °C for 30 min.

Vine tea samples were collected during processing, immediately frozen in liquid nitrogen, transported, vacuum freeze-dried, and used for volatile profiling, physicochemical attribute assessment, and metabolomics analysis. All samples originated from a single harvest season, geographical origin, and production batch, with three technical replicates per sample. These replicates capture instrument and preparation variability but not biological or processing variation; therefore, the findings reflect the specific production lot and require multi-batch validation for broader generalization.

#### 2.1.2. Chemical Standards

Standards for dihydromyricetin, myricitrin, and taxifolin were obtained from Chengdu Ref Medic Biotech Co., Ltd. (Chengdu, China). Ethyl caprate (analytical reagent, internal standard) was purchased from Sigma-Aldrich (Shanghai, China). Distilled water was obtained from Watsons (Guangzhou, China). SPME fiber holders and manual sampling accessories were from Supelco (Bellefonte, PA, USA). CAR/DVB/PDMS (50/30 μm, 2 cm) and DVB/Carbon WR/PDMS (80 μm) fibers were obtained from Supelco (Bellefonte, PA, USA).

### 2.2. Determination of Vine Tea Color Qualities

Color was assessed in CIELab space (*L*, *a*, *b*): *L* (0 = black, 100 = white), *a* (negative = green, positive = red), and *b* (negative = blue, positive = yellow). Dry leaves and brew were measured using a spectrophotometer (CM-5, Konica Minolta, Shanghai, China), with distilled water as the reference [15]. For dried leaves and infused leaves, the sample was placed in a quartz cuvette and measured in reflectance mode against a standard white plate (calibration tile). Each measurement was performed in triplicate. For brew, 2.0 g of dried vine tea was infused with 150 mL of boiling water for 4 min in a covered glass beaker, followed by filtration. The cooled filtrate was measured in transmittance mode using a 10 mm path-length cuvette, with deionized water as blank.

### 2.3. Determination of Physicochemical Qualities

Moisture content was determined by oven drying at 120 °C. Water extract content was determined according to GB/T 8305-2002 [7]. Total polyphenols were quantified by the iron tartrate colorimetric method [16]. Free amino acids were measured using the ninhydrin colorimetric method, and soluble sugars by the anthrone colorimetric method. Flavonoids (dihydromyricetin, taxifolin, myricitrin and myricetin) [17] were quantified by HPLC using a C18 column (4.6 × 250 mm, 5 μm) with methanol (A)—a 0.1% phosphoric acid (B) gradient. Detection wavelengths were 290 nm (0–21 min) and 255 nm (21–30 min); the flow rate was 1.0 mL/min, the column temperature was 30 °C, and the injection volume was 10 μL [18,19]. The wavelength was switched at 21 min because taxifolin and dihydromyricetin exhibit maximum UV absorption at approximately 290 nm, whereas myricitrin and myricetin show stronger absorption at 255 nm; the switch thus allowed each compound to be detected at its optimal wavelength for maximum sensitivity.

### 2.4. Non-Targeted GC-MS Metabolomics for Relative VOC Profiling and Differential Abundance Analysis

Non-targeted volatile profiling was performed on five independent replicates per processing stage (fresh leaves, fixation, rolling, stacking, drying). Approximately 1 g of sample was placed in a headspace vial with 20 μL of internal standard (ethyl decanoate, 10 mg/L). Volatiles were extracted using an automatic headspace sampler under the following conditions: equilibration at 60 °C for 15 min at 250 r/min, followed by headspace extraction with a DVB/Carbon WR/PDMS (50/30 μm) fiber at 60 °C for 30 min, and thermal desorption at 260 °C for 5 min. The fiber was conditioned between runs to minimize carryover.

GC-MS analysis was conducted on an Agilent 7890-5975 system equipped with an HP-5MS UI column (30 m × 0.25 mm × 0.25 μm) (Supelco, Bellefonte, PA, USA). High-purity helium (1.0 mL/min) served as carrier gas, with a split injection ratio of 25:1. The oven program was 50 °C for 5 min, which was ramped to 230 °C at 7 °C/min (hold 3 min) and then to 320 °C at 40 °C/min (hold 5 min). The ion source and quadrupole were set at 240 °C and 160 °C, respectively, with electron impact ionization at 70 eV and a mass range of *m*/*z* 50–500.

Raw data were converted to .abf format and processed using MSDIAL 4.60 for peak finding, alignment, and integration. Metabolites were identified by matching mass spectra and retention indices against the NIST2020 library, with all compounds assigned MSI Level 2 (putatively annotated).

The 60 °C extraction temperature, while standard for tea volatiles, is at the upper end of the range recommended for sensory fidelity-focused analyses (30–45 °C). This condition may bias toward higher-boiling compounds and underrepresent highly volatile aroma contributors, though comparative conclusions remain valid as all samples were treated identically. A single internal standard was used for semi-quantification assuming equivalent detector response, as authentic standards for all 280 compounds were unavailable. The resulting data are, therefore, relative rather than absolute, and statistical outputs (PLS-DA, VIP, fold-change) should be interpreted accordingly, with discriminant compounds viewed as candidates requiring targeted validation.

### 2.5. Aroma-Oriented VOC Quantification, Identification, and ROAV Analysis for Odor Intensity Evaluation

Volatile compounds were analyzed by HS-SPME coupled with GC–MS (Agilent 7890A GC, 5975C MSD). For each analysis, 3.0 g of dried tea leaves were placed in a 100 mL vial with 30 mL of boiling water and 20 μL of ethyl caprate (internal standard). After equilibration at 50 °C for 10 min, volatiles were extracted using a CAR/DVB/PDMS fiber (preconditioned at 250 °C for 10 min between runs) and separated on a DB-5MS column (60 m × 0.25 mm × 0.32 μm). The oven program was 50 °C (5 min), which was ramped to 180 °C at 3 °C/min (hold 2 min) and then to 250 °C at 10 °C/min (hold 3 min). Helium carrier flow was 1.0 mL/min. MS detection was performed in EI mode (70 eV) over a scan range of *m*/*z* 45–400. Compounds were identified using the NIST20 library, retaining only matches with spectral score ≥ 700, and relative contents were normalized to the internal standard.

A single internal standard (ethyl caprate) was used for semi-quantification, assuming unity response factors for all analytes due to the large number of identified compounds (n > 100) and the commercial unavailability of authentic standards. The resulting data are, therefore, relative estimates suitable for cross-sample comparisons rather than absolute concentrations, as detector response factors can vary substantially across compound classes (potentially by an order of magnitude). All numerical values should be interpreted as estimates preserving ordinal relationships among samples.

### 2.6. Preliminary Aroma Activity Screening

Relative odor activity values (ROAVs) were calculated as the ratio of compound concentration to its odor detection threshold (ODT) [20,21]. Concentrations were estimated as concentration = (compound peak area/ethyl caprate peak area) × ethyl caprate concentration (μg/L); ODT values were obtained from the literature.

To obtain an exploratory indication of volatile compounds that may contribute to aroma differences among samples, a modified relative odor activity value (ROAV) approach was applied. It must be emphasized that this calculation is exploratory and semi-quantitative in nature, given that the underlying concentrations are relative estimates derived from single-internal-standard quantification. The ROAV was calculated using the following formula:

$$ROAVi=\frac{Ci}{Ti}\times \frac{T^{\mathrm{std}}}{C^{\mathrm{std}}}\times 100$$

where Ci and Ti are the semi-quantitative concentration and odor detection threshold (ODT) of compound "i", respectively, and C^std^ and T^std^ are those of the compound with the highest OAV (set to 100). This normalized version allows direct comparison of relative aroma contributions across compounds. This formulation follows the normalized ROAV convention. The resulting ROAVi values should not be interpreted as accurate measures of sensory impact but rather as a preliminary screening tool to generate hypotheses about which compounds may warrant further targeted investigation using authentic standards and odor threshold validation.

### 2.7. GC–O Analysis

GC-O analysis was performed alongside the HS-SPME-GC–MS procedure described in Section 2.5, using the same column and oven program. Injector and transfer line temperatures were 230 °C and 260 °C, respectively, with nitrogen (99.99%) as carrier gas. The column effluent was split 1:1 between the MS and an olfactory detection port (ODP3, Gerstel, Nordrhein-westfalen, Germany), with the sniffing port transfer line maintained at 260 °C and humidified nitrogen added to prevent nasal dryness [20].

Five trained assessors from the Fruit and Tea Research Institute (Hubei Academy of Agricultural Sciences), with prior sensory evaluation experience, participated in the GC-O analysis. Training followed a five-step procedure covering method introduction, odor vocabulary and intensity scale familiarization (four-point scale: 1 = weak to 4 = extremely strong), and practice with reference standards and vine tea samples; only assessors demonstrating consistent performance in three consecutive sessions were included [8].

### 2.8. Statistical Analyses

All measurements included at least three technical replicates, with the results reported as mean ± standard error. One-way ANOVA followed by Duncan’s multiple range tests was used for group comparisons. PLS-DA was performed in MetaboAnalyst 5.0; data were preprocessed by removing variables with >60% missing values, followed by median normalization, logarithmic transformation, and autoscaling. Graphs were prepared using GraphPad Prism v8.0.2.

## 3. Results and Discussion

### 3.1. Impact of Processing Procedure on Vine Tea Color Qualities

**Figure 2:**
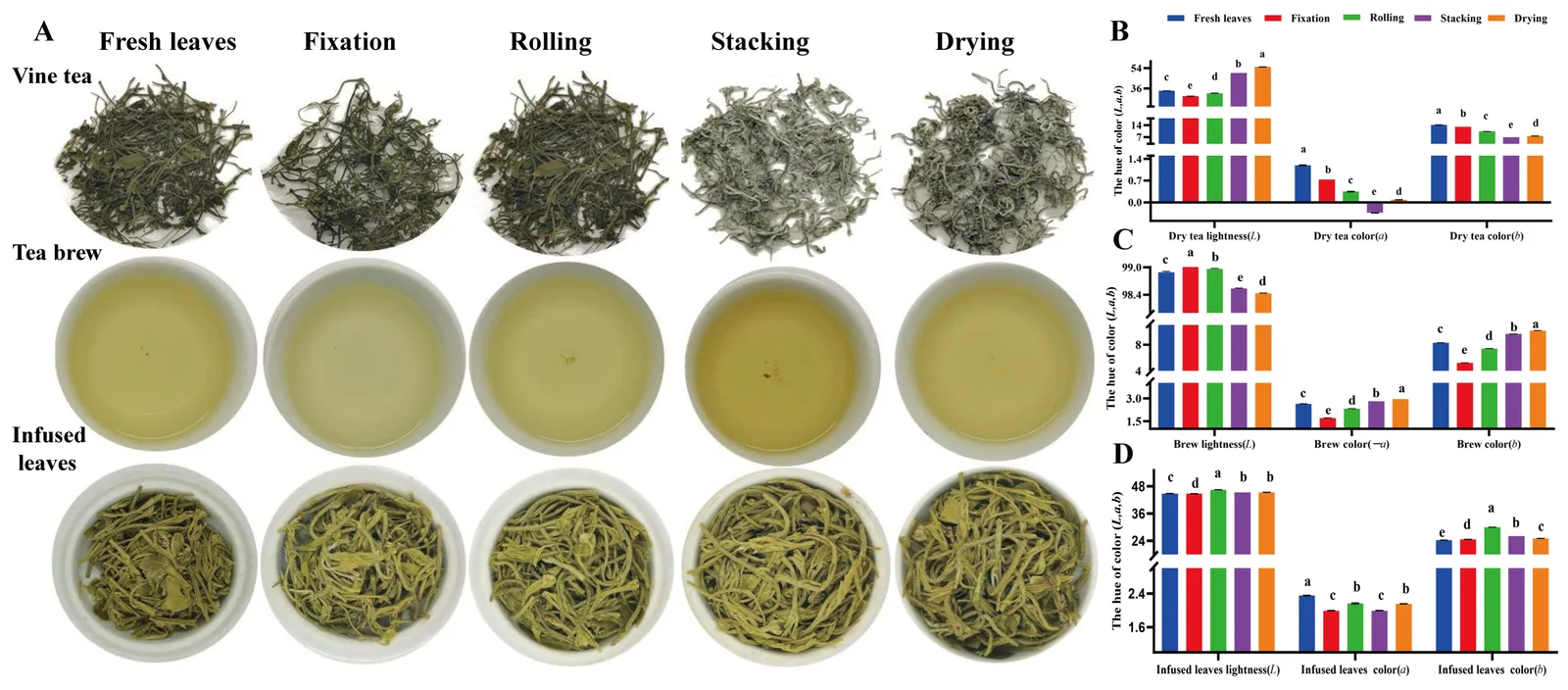
(**A**) Appearance of vine tea, brew, and infused leaves during processing. (**B**–**D**) Quantification of hue (*L*, *a*, *b*) for dry tea, brew, and infused leaves, respectively. Columns labeled ‘a’, ‘b’, ‘c’, ‘d ’, and ‘e’ had significant differences (*p* < 0.05) from each other.

Processing markedly altered vine tea color (Figure 2). Across processing stages, the *L* value of dry tea increased overall, with a pronounced rise during stacking (*L* increased by 55.90%), while *a* and *b* decreased by 197% and 33.37%, respectively, indicating progressive whitening and yellowing of the dry leaves during stacking. Conversely, the fixation stage produced the highest brew brightness (highest *L*) and the greenest brew and infused leaves. After fixation, continued processing produced darker, redder brews: *L* declined, and both *a* and *b* increased as processing proceeded. The whitening of dry leaves may be associated with the surface deposition of phenolic compounds, such as dihydromyricetin, during moisture loss; however, this interpretation is speculative and requires further investigation. The browning of infusions may similarly result from pigment oxidation, though the underlying mechanisms are not yet established. These observations are consistent with previous reports of processing-driven chemical changes and color shifts in vine tea and related products [16,22,23] and are plausibly explained by enzymatic browning, pigment oxidation, and Maillard reactions occurring during processing [15,24]. Processing also alters cellular structure, which in turn modifies chemical composition [25,26].

### 3.2. Impact of Processing Procedure on Vine Tea Non-Volatile Chemicals

**Figure 3:**
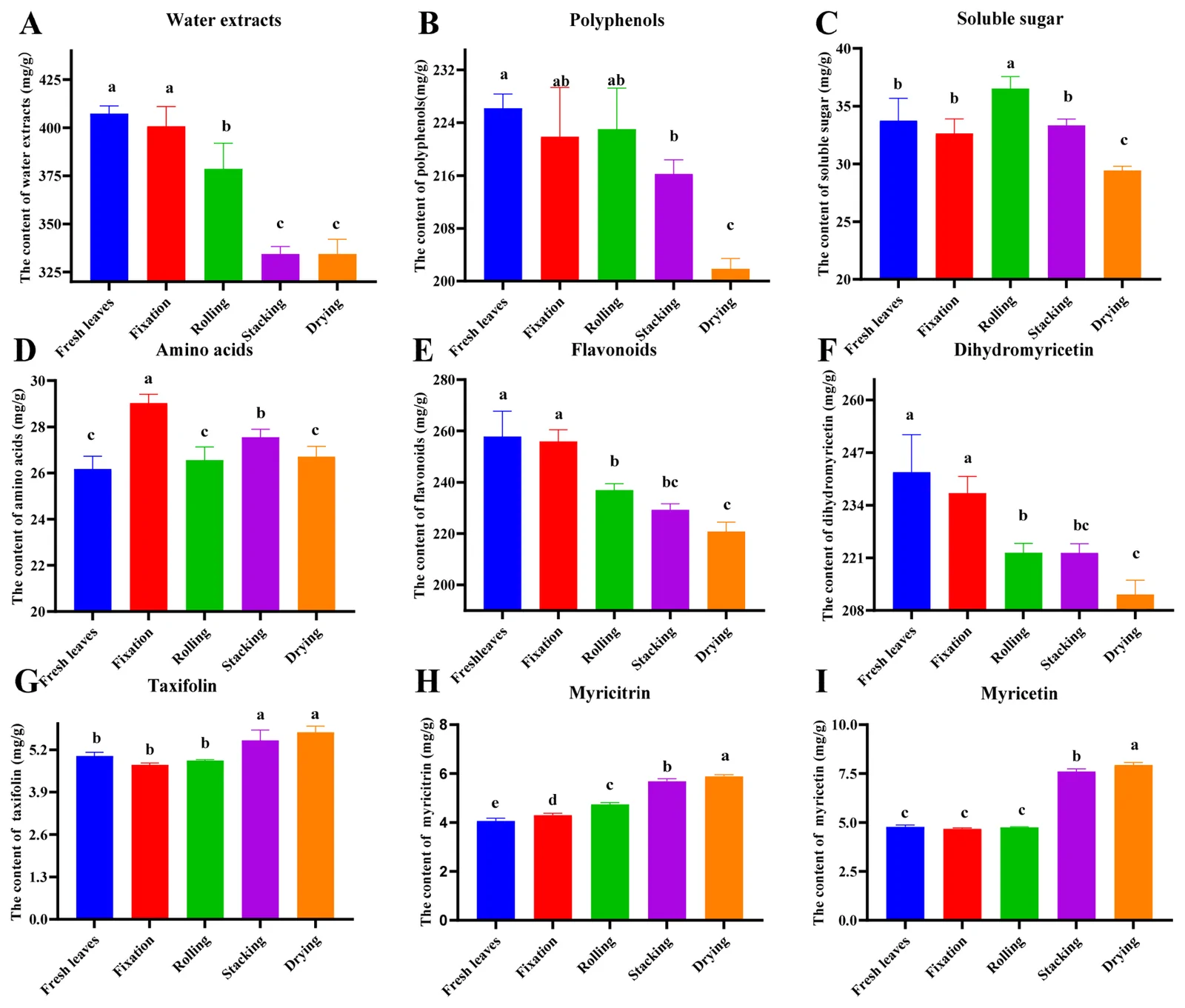
Changes in main physicochemical quality indices during vine tea processing. (**A**–**I**) Water extracts, polyphenols, soluble sugars, amino acids, flavonoids, dihydromyricetin, taxifolin, myricitrin, and myricetin. Columns labeled ‘a’, ‘b’, ‘c’, ‘d’, and ‘e’ had significant differences (*p* < 0.05) from each other.

Processing induced substantial changes in non-volatile composition (Figure 3). We quantified water extracts (Figure 3A), polyphenols (Figure 3B), soluble sugars (Figure 3C), amino acids (Figure 3D), total flavonoids (Figure 3E), and individual flavonoids—dihydromyricetin (Figure 3F), taxifolin (Figure 3G), myricitrin (Figure 3H), and myricetin (Figure 3I). Overall, water extracts, polyphenols, total flavonoids, and dihydromyricetin decreased during processing, whereas soluble sugars and amino acids rose initially and then declined. These compositional shifts have direct implications for taste and aroma.

Water extracts—comprising flavonoids, amino acids, and sugars and linked to antioxidant activity and potential food industry applications [27]—declined progressively, with the steepest losses during rolling and stacking; stacking yielded the lowest water-extract content, suggesting that it is a key step for flavor formation, in agreement with the findings of Qi et al. [26]. Polyphenols, which have been reported to be associated with bitterness and astringency [28,29], decreased across processing, likely due to oxidation and thermal degradation during fixation and stacking. Soluble sugars, often considered important for infusion sweetness [22,26], peaked after rolling and reached a minimum after drying; the rolling-associated increase in certain volatiles may be partly attributable to glycoside hydrolysis, and the post-drying decline may involve Maillard and caramelization reactions; however, these mechanistic assignments remain speculative and require targeted verification.

Amino acids contributed to the overall flavor through various taste modalities: glutamic and aspartic acids impart umami, while other amino acids may be associated with sweetness, bitterness, or astringency, depending on their side-chain structures and concentrations [30,31]; free amino acids were highest after fixation and remained relatively high after stacking. The observed changes in amino acid levels are experimentally demonstrated, while the underlying mechanisms (e.g., protein hydrolysis via thermal denaturation and decarboxylation/oxidation pathways) are inferred from previous reports [16] and should be considered hypothesis-generating. Similarly, the depletion of certain amino acids during rolling, stacking, and drying is consistent with potential Maillard reactions [26], but direct evidence for this pathway in vine tea processing is not yet available.

Flavonoids, the principal bioactive metabolites in *Nekemias grossedentata* [15,32], showed an initial increase followed by a decline (Figure 3E), consistent with thermal and mechanical facilitation of glycoside hydrolysis and subsequent conversion or degradation during stacking and drying. Dihydromyricetin declined steadily and was lowest after drying (Figure 3F), likely reflecting thermal decomposition and oxidative loss. In contrast, taxifolin, myricitrin, and myricetin increased during processing, especially during stacking—consistent with hydrolysis-driven release or transformation of precursor compounds.

### 3.3. Impact of Processing Procedures on VOCs

#### 3.3.1. Volatile Organic Compounds (VOCs) Across Processing Stages

**Figure 4:**
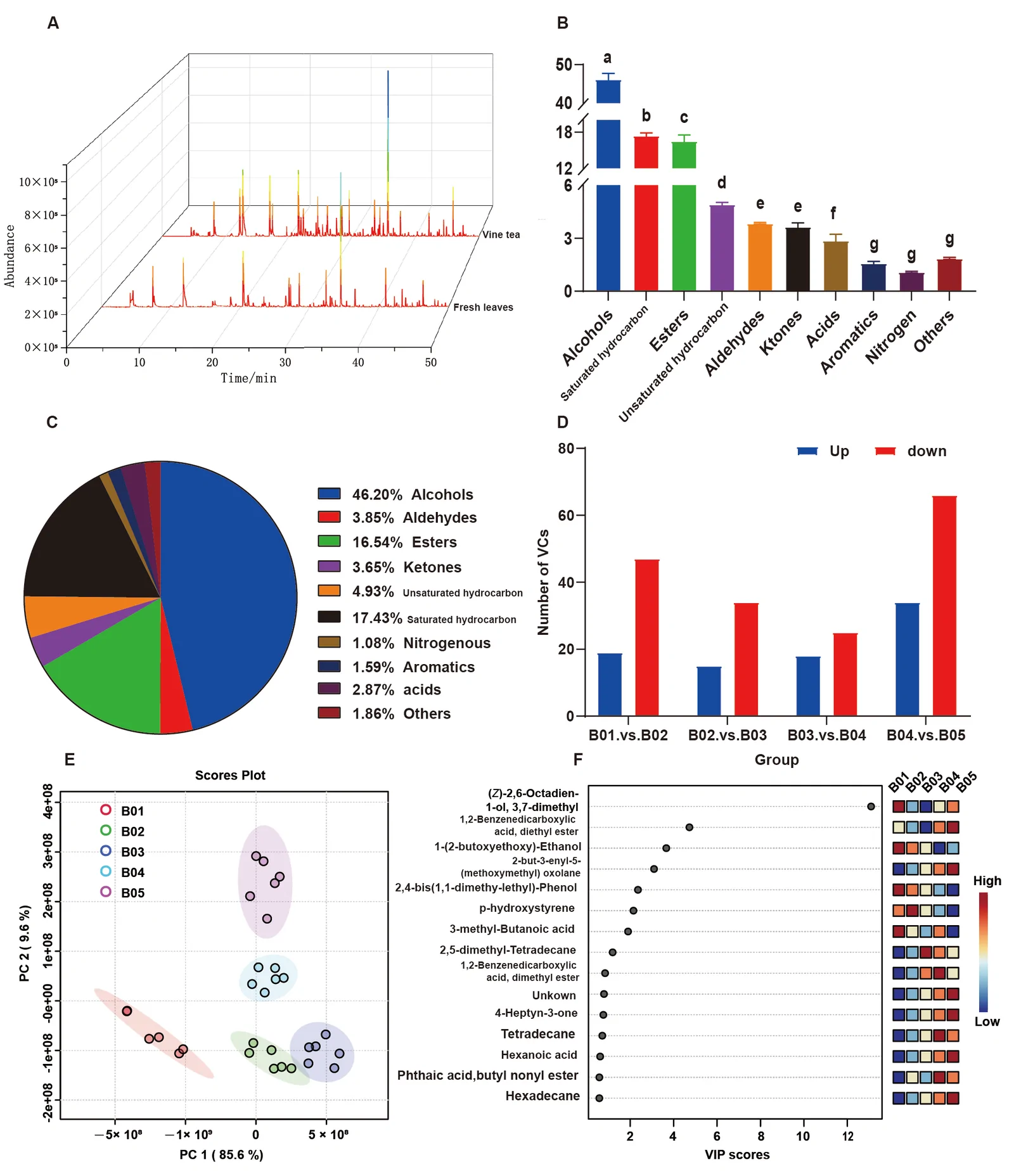
Analysis of differentially abundant volatile organic compounds (VOCs) during vine tea processing. (**A**) The chromatogram of fresh leaves and vine tea samples. (**B**,**C**) Classification and proportional content of volatile organic compounds. (**D**) The number of VOCs with fold change ≥1.50 (up-regulated) or ≤0.67 (down-regulated) (*p*< 0.01) at each stage. (**E**) PLS-DA score plot. (**F**) Top 15 volatile organic compound components by VIP value. B1, fresh leaves; B2, fixed leaves; B3, rolled leaves; B4, stacked leaves; B5, dried leaves. Columns labeled ‘a’, ‘b’, ‘c’, ‘d’, ‘e’, ‘f’, and ‘g’ had significant differences (*p* < 0.05) from each other.

Based on the chromatogram and mass spectrum shown in the Results (Figure 4A), HS-SPME-GC-MS classified 280 identified volatile organic compounds into ten chemical families (Figure 4B). Alcohols were the dominant class (46.20%), significantly exceeding other groups (*p* < 0.05), followed by saturated hydrocarbons (17.43%) and esters (16.54%); together, alcohols, saturated hydrocarbons, and esters accounted for >80% of volatiles, identifying them as the principal volatile pools in vine tea (Figure 4C).

To elucidate the impact of processing steps on volatile organic compounds, compounds exhibiting significant differences (*p* < 0.01) with fold changes ≥1.50 or ≤0.67 were identified at each stage (Figure 4D); the threshold was selected because a 1.5-fold change represents a ≥50% alteration in abundance, commonly regarded as biologically meaningful in metabolomics studies. The reciprocal 0.67 cutoff was applied to maintain symmetrical criteria for down-regulated compounds. The most substantial changes in aroma components occurred during drying (Figure 4D); 100 volatiles changed significantly during drying (34 up-regulated, 66 down-regulated), followed by fixation (19 up-regulated, 47 down-regulated) and then by rolling and stacking. The large number of differential volatile organic compounds observed after drying further underscores the critical role of this step in determining vine tea quality; the fixation process also exerted a considerable influence on aroma development.

PLS-DA (Figure 4E) captured 95.2% of the variance in the first two components and clearly discriminated between processing stages, confirming substantial biochemical rearrangements across procedures. Variable importance in projection (VIP) scores identified 15 VOCs with VIP > 1 (Figure 4F), marking them as the most influential contributors to the processed tea’s aroma and physicochemical profile.

Tracking the dynamics of different volatile organic compounds across fresh leaves (B1), fixation (B2), rolling (B3), stacking (B4), and drying (B5) (Table 1), the contents of alcohols, acids, and others were highest in fresh leaves and decreased progressively during processing, reaching their lowest levels after drying. This trend suggests that enzyme inactivation and mechanical manipulation either suppressed the formation of these compounds or accelerated their volatilization. In contrast, aldehydes, ketones, aromatics, and unsaturated hydrocarbons peaked after rolling and then declined with subsequent processing steps, indicating that they were formed or released during mechanical treatment and subsequently reduced by thermal exposure. Esters gradually increased throughout processing and reached their maximum after drying, which implies that the thermal step facilitates ester formation, implying that thermal steps favor ester generation or concentration.

#### 3.3.2. Effects of Processing Steps on Differential VOCs of Vine Tea

**Figure 5:**
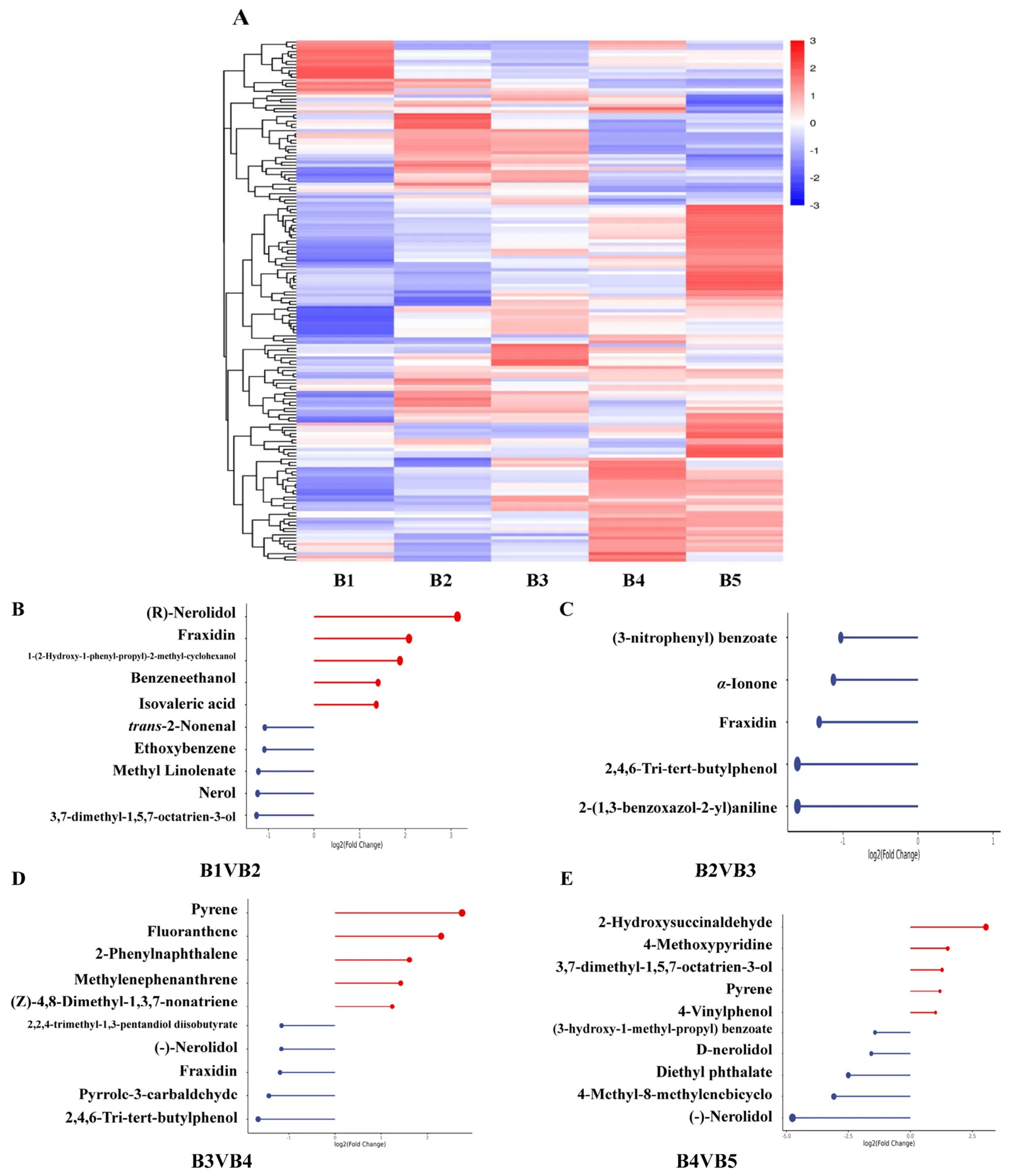
Multivariate statistical analysis of volatile organic compounds during the manufacturing of vine tea. (**A**) Heatmap of possible key differential volatile components during the processing of vine tea in fresh leaves (B1), fixed leaves (B2), rolled leaves (B3), stacked leaves (B4), and dried leaves (B5). Visualization of differentially accumulated volatile organic compounds: (**B**) B1 vs. B2, (**C**) B2 vs. B3, (**D**) B3 vs. B4, (**E**) B4 vs. B5. The red and blue represent increase and decrease respectively.

Given the semi-quantitative nature of the VOC data, the multivariate statistical outputs—particularly VIP scores and differential abundance rankings—should be interpreted as trend indicators and hypothesis-generating tools rather than precise quantitative conclusions. These results provide prioritization for future targeted validation with authentic standards. HCA, PCA, and PLS-DA were applied to the non-targeted metabolomics dataset to visualize similarities and differences among processing stages and to identify differential volatile organic compounds (DVOCs) [20]. HCA based on Euclidean distance clearly separated the five processing stages (Figure 5A), with heatmap color blocks (blue → red) reflecting low → high compound abundance across samples.

PCA compressed the data into two principal components that captured the bulk of variation (PC1 = 43.77%, PC2 = 39.31%), and the score plots showed distinct clustering of fresh leaves (B1), fixed leaves (B2), rolled leaves (B3), stacked leaves (B4), and dried leaves (B5) (Figure S1). To achieve higher class separation and identify predictive markers, supervised PLS-DA was used for pairwise stage comparisons (B1 vs. B2, B2 vs. B3, B3 vs. B4, and B4 vs. B5). The PLS-DA models exhibited strong predictive ability (Q^2^ = 0.98–1.00; Q^2^ > 0.7 indicates good predictivity), and permutation tests returned intercepts of the Q^2^ regression line < 0, supporting model reliability.

Pairwise differential analysis revealed stage-specific shifts in volatile organic compounds (Figure S1). After fixation (B1), the top five up-regulated DVOCs were (R)-nerolidol, fraxidin, 1-(2-hydroxy-1-phenyl-propyl)-2-methylcyclohexanol, benzeneethanol, and isovaleric acid; major down-regulated compounds included *trans*-2-nonenal, ethoxybenzene, methyl linolenate, nerol, and 3,7-dimethyl-1,5,7-octatrien-3-ol (Figure 5B). The increase in phenylpropanoid metabolites following fixation may reflect a stress response to rapid water loss and heat shock, potentially involving PAL activity. However, this interpretation is speculative and based on correlative observations; direct measurements of enzyme activity or gene expression would be needed to confirm the proposed mechanism.

During the rolling stage, the top five down-regulated differential volatile compounds (DVOCs) included 3-nitrophenyl benzoate, *α*-ionone, fraxidin, 2,4,6-tri-tert-butylphenol, and 2-(1,3-benzoxazol-2-yl) aniline (Figure 5C). After fixation, leaves were cooled until pliable, then rolled. Mechanical disruption promoted volatilization and loss of existing volatiles, especially moderately volatile or surface-bound compounds, leading to a decrease in their measured abundance.

During stacking, the top up-regulated DVOCs were pyrene, fluoranthene, 2-phenylnaphthalene, methylenephenanthrene, and (*Z*)-4,8-dimethyl-1,3,7-nonatriene. Down-regulated DVOCs included 2,2,4-trimethyl-1,3-pentandiol diisobutyrate, (-)-nerolidol, fraxidin, pyrrole-3-carbaldehyde, and 2,4,6-tri-tert-butylphenol (Figure 5D). The predominance of up-regulated volatiles after stacking suggests that low-temperature and moisture-controlled conditions during this stage may promote the generation of new aroma species through non-enzymatic or enzyme-mediated transformations, potentially contributing to consumer acceptance [26].

Drying caused the largest net change in volatile composition. The top up-regulated DVOCs were 2-hydroxysuccinaldehyde, 4-methoxypyridine, 3,7-dimethyl-1,5,7-octatrien-3-ol, pyrene, and 4-vinylphenol. Down-regulated species included (3-hydroxy-1-methyl-propyl) benzoate, D-nerolidol, diethyl phthalate, 4-methyl-8-methylenebicyclo, and (-)-nerolidol (Figure 5E). During drying, the number and magnitude of up-regulated DVOCs exceeded those of down-regulated ones, consistent with the observed net increase in total peak area (Figure 2D). This may reflect concurrent thermal degradation, volatilization losses, and secondary formation reactions that collectively reconfigured the volatile profiles.

#### 3.3.3. Effects of Processing Steps on the Aroma Intensity of Major Vine Tea Components

Among the analytical approaches employed in this study, GC-O analysis provides the most methodologically robust evidence for aroma activity, as it constitutes a direct sensory measurement at the detector port that is independent of concentration quantification and response factor assumptions. These GC-O findings form the sensory foundation for the subsequent discussion of process-induced aroma changes.

The present study distinguishes itself from previous vine tea volatile research in three key aspects. First, non-targeted metabolomics and machine learning approaches had been employed for volatile screening [17,26,33,34], while the GC-O analysis provided direct sensory evidence that is independent of concentration quantification. Second, unlike studies that characterize volatile compounds at discrete time points, we systematically traced the fate of aroma-active compounds across each processing step and linked them to their respective formation pathways. Third, whereas previous GC-O studies on vine tea focused on grade comparison or static characterization, our study is the first to apply GC-O across the entire processing chain to identify processing-specific aroma markers.

Vine tea processing involves thermochemical transformations that dynamically alter volatile organic compounds [33]. To elucidate the evolution of key VOCs, relative odor activity values (ROAVs) were quantified across stages, and odor descriptors, intensities, and ROAVs were analyzed using GC-O. Ten key aroma-active compounds exhibited ROAVs ≥ 1% (Table 2). *β*-ionone showed the highest ROAV, followed by *β*-myrcene (peppery, balsamic, spicy) and nonanal (sweet).

In fresh leaves, dominant volatiles included *β*-ocimene, geranyl isovalerate, geraniol, nonanal, and hexanal. During fixation, the ROAV of most compounds decreased, likely due to thermal evaporation and enzyme inactivation. Rolling caused minor fluctuations. However, during stacking, the ROAV of hexanal, heptanal, sulcatone, *β*-myrcene, *β*-ocimene, undecanal, nonanal, decanal, *α*-citral, and *β*-ionone increased markedly. After drying, *β*-ionone exhibited the highest ROAV (100%), followed by *β*-ocimene (17.31%), nonanal (14.97%), hexanal (5.87%), and *β*-myrcene (4.83%). *β*-ionone, a known contributor to fruity/floral notes in teas [30], remained most prominent, while *β*-ocimene imparted a floral scent. Nonanal and hexanal-fatty acid derivatives with flowery/honey-like notes, and *β*-myrcene (sweet, balsamic), all contributed to the distinctive aroma profile [33]. Regarding potential formation pathways [8], the following potential formation pathways are speculative and based solely on volatile metabolite correlations. The accumulation of hexanal and nonanal during stacking and drying could arise from fatty acid degradation (LOX pathway or thermal auto-oxidation). The increase in *β*-ionone during drying may reflect carotenoid cleavage (CCDs or heat-induced degradation). The decrease in monoterpenes during fixation might be due to thermal evaporation, though glycosidic hydrolysis in earlier stages could also contribute. These hypotheses require direct experimental confirmation.

Although the ROAV has been widely used to estimate the contribution of individual volatiles, it relies on concentration and threshold values obtained from simplified matrices and does not reflect real-time sensory interactions. In contrast, GC-O directly measures the olfactory perception of compounds as they elute from the column under conditions matching the actual sample matrix. Therefore, in this study, priority is given to compounds that were consistently detected by GC-O with moderate to high aroma intensity (AI ≥ 2), regardless of their calculated ROAVs. Collectively, the GC-O data consistently identified *β*-ionone, *β*-ocimene, nonanal, hexanal, and *β*-myrcene as the most salient aroma-active compounds across samples, providing direct sensory evidence that takes priority over the semi-quantitative abundance estimates and the exploratory ROAV rankings. These GC-O findings form the sensory foundation for the subsequent discussion of process-induced aroma changes.

### 3.4. Hypothesized Formation Pathways of Aroma-Active Compounds

The GC-MS-O results showed that aroma-active compounds including *β*-ionone, *β*-ocimene, nonanal, hexanal, and *β*-myrcene changed dramatically during drying and fixation. To explore potential formation pathways, we mapped the identified volatiles to the Kyoto Encyclopedia of Genes and Genomes (KEGG) reference pathways. However, given that our data are based solely on volatile profiling without direct enzymatic or transcriptomic evidence, this analysis is exploratory and hypothesis-generating. The following pathways, terpenoid biosynthesis, carotenoid cleavage, and fatty acid degradation, have been implicated in aroma formation [34].

**Figure 6:**
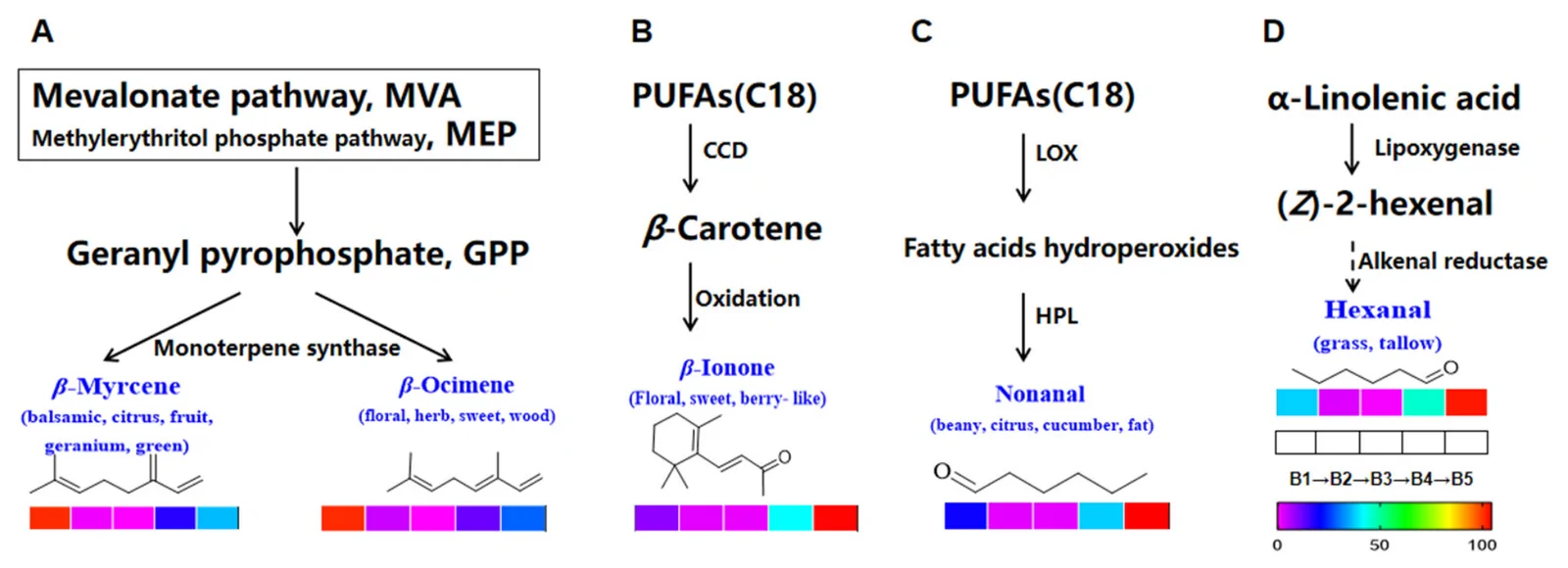
Metabolic transformation of the major flavor-active compounds during manufacture. (**A**) *β*-Ocimene, *β*-Myrcene; (**B**) *β*-Ionone; (**C**) Nonanal; (**D**) Hexanal.

Monoterpenes such as *β*-ocimene and *β*-myrcene are known intermediates of the terpenoid biosynthesis pathway, originating from geranyl pyrophosphate via terpene synthases (TPSs) in other plant systems (Figure 6A). Their decrease during processing, particularly at fixation, may be attributable to thermal evaporation, although this remains speculative in the absence of direct measurements. *β*-ionone is a C13 apocarotenoid that can be generated by oxidative cleavage of *β*-carotene, catalyzed by carotenoid cleavage dioxygenases (CCDs) in previously characterized pathways (Figure 6B) [34]; its increase during drying could hypothetically reflect enhanced cleavage activity. The hexanal and nonanal aldehydes may arise via the lipoxygenase (LOX)–hydroperoxide lyase (HPL) pathway from linoleic and linolenic acids (Figure 6C,D) [36], and the increase observed after drying is consistent with previous reports [23], suggesting that drying conditions may promote the formation of these aliphatic aldehydes.

Based on these findings and supporting evidence from recent studies [5,37,38,39,40,41], the formation of aroma-active compounds in vine tea could potentially involve multiple biochemical pathways. Specifically, the increase in hexanal and nonanal may be associated with fatty acid degradation, which might contribute to herbaceous and green odor characteristics [18]. In contrast, the elevated level of *β*-ionone during the drying stage could be related to carotenoid cleavage, potentially contributing to sweet or fruity notes [8].

## 4. Conclusions

This study was conducted on a single production batch (one harvest, one location, one processing run) with technical replicates only. The findings, including VOC profiles, PLS-DA discrimination patterns, and aroma activity rankings, are representative of this specific lot and should not be generalized to vine tea production as a whole without multi-batch, multi-season, and multi-location validation incorporating biological replicates. GC-O data, as direct sensory measurements, provide the most robust evidence for aroma-active compound identification within this defined scope.

In this study, we employed color analysis, physicochemical characterization, untargeted metabolomics, HS-SPME-GC-MS, ROAV, and GC-O to comprehensively evaluate the effects of processing on vine tea quality. Throughout processing, the *L* value progressively increased, while *a* and *b* values decreased, indicating a lightening of color and reduced chromatic intensity. Concurrently, the contents of water extract, polyphenols, flavonoids, and dihydromyricetin decreased significantly, whereas soluble sugars and amino acids showed an initial increase followed by a decrease. A total of 280 volatile organic compounds were identified, among which 14 compounds were confirmed as aroma-active via ROAV and GC-O analyses. Non-volatile constituents were dominated by polyphenols, flavonoids, and dihydromyricetin, while alcohols were the predominant volatile class. Notably, *β*-ionone, *β*-myrcene, nonanal, and hexanal exhibited higher ROAVs and accumulated predominantly during processing, contributing substantially to the characteristic flavor of vine tea. Furthermore, fixation and drying were identified as critical steps for vine tea aroma quality formation, serving as key transition points for most volatile organic compounds. Based on the correlation analysis between VOC profiles and sensory attributes, non-enzymatic degradation of fatty acids is proposed as a potential contributor to the formation of sweet and fruity aroma notes.

These findings imply that regulating dihydromyricetin and key aroma components during drying and fixation is essential—specifically to minimize the loss of bioactive compounds and to enhance desirable floral and fruity aroma notes in the final product. However, the present study did not explore the interactive effects of specific processing parameters within these critical steps. Future research should systematically optimize drying and fixation conditions—for example, using response surface methodology—to balance the retention of bioactive compounds with the formation of key volatiles. Additionally, controlled experiments employing heat-treated samples are needed to distinguish the relative contributions of enzymatic versus non-enzymatic reactions during these stages. This study provides insights that may inform future efforts toward processing optimization and quality improvement of vine tea. Furthermore, future work should include sensory analysis with a trained panel to validate the contribution of key taste and aroma compounds to the overall sensory quality of vine tea.

## Figures and Tables

**Table 1:** Changes in relative contents of different compounds across processing.

Compounds	Fresh Leaves	Fixation	Rolling	Stacking	Drying
Alcohols	61.99 ± 0.82 a	51.67 ± 0.57 b	47.15 ± 1.78 d	48.91 ± 1.67 c	46.4 ± 1.57 c
Aldehydes	2.23 ± 0.17 c	3.69 ± 0.11 a	3.88 ± 0.35 a	3.15 ± 0.09 b	3.85 ± 0.05 a
Esters	8.67 ± 0.14 e	9.33 ± 0.09 d	10.97 ± 0.21 c	12.56 ± 0.39 b	16.54 ± 0.99 a
Ketones	1.73 ± 0.19 c	3.24 ± 0.18 b	4.05 ± 0.77 a	3.57 ± 0.35 ab	3.65 ± 0.22 ab
Unsaturated hydrocarbons	4.23 ± 0.1 c	5.87 ± 0.22 a	5.81 ± 0.4 a	5.13 ± 0.18 b	4.93 ± 0.11 b
Nitrogenous	0.59 ± 0.03 c	0.91 ± 0.05 b	1.07 ± 0.10 a	0.94 ± 0.05 b	1.08 ± 0.05 a
Aromatics	1.39 ± 0.04 c	1.73 ± 0.05 b	1.93 ± 0.1 a	1.43 ± 0.07 c	1.59 ± 0.10 c
Acids	4.12 ± 0.50 a	2.9 ± 0.33 c	3.14 ± 0.43 bc	3.61 ± 0.21 b	2.87 ± 0.35 c
Saturated hydrocarbons	10.8 ± 0.48 d	16.82 ± 0.55 c	19.17 ± 1.02 a	18.05 ± 0.45 b	17.43 ± 0.44 bc
Others	4.02 ± 0.32 ab	16.82 ± 0.56 a	3.78 ± 0.26 b	2.72 ± 0.04 c	1.86 ± 0.06 d

Distinct letters (a–e) within the identical row signify significant variations among the vine tea processing (*p* < 0.05).

**Table 2:** Changes in aroma intensity and ROAVs of vine tea across different processing stages.

Compounds	RI ^a^	Aroma Description ^b^	ODT (μg/kg) [16,35] ^c^,^d^	Aroma Intensity					ROVA/%				
				B1	B2	B3	B4	B5	B1	B2	B3	B4	B5
*α*-Citral	1276	Citrus, lemon	12.0	1.0 ± 0.1 b	1.0 ± 0.1 b	1.0 ± 0.1 b	1.5 ± 0.1 a	1.0 ± 0.1 b	0.05 ± 0.00 c	0.06 ± 0.00 b	0.05 ± 0.00 c	0.08 ± 0.00 a	0.08 ± 0.00 a
Decanal	1487	Tallow	0.3	2.0 ± 0.2 a	2.0 ± 0.2 a	2.0 ± 0.2 a	2.0 ± 0.2 a	2.0 ± 0.2 a	1.36 ± 0.00 b	1.34 ± 0.00 c	1.24 ± 0.00 e	1.33 ± 0.00 d	1.37 ± 0.00 a
Geranyl Isovalerate	1105	Oily, minty, floral	4.6	2.0 ± 0.2 a	2.0 ± 0.2 a	2.0 ± 0.2 a	1.0 ± 0.2 b	2.0 ± 0.2 a	27.68 ± 0.83 a	6.66 ± 0.00 b	5.97 ± 0.02 c	3.98 ± 0.04 d	3.90 ± 0.08 e
Heptanal	1182	Sweet, chestnut-like, grass	3.0	1.0 ± 0.1 b	1.0 ± 0.1 b	1.0 ± 0.1 b	1.5 ± 0.1 a	1.0 ± 0.1 b	1.65 ± 0.00 c	1.20 ± 0.00 d	1.15 ± 0.00 e	1.73 ± 0.00 b	2.04 ± 0.00 a
(*E*)-2-Heptenal	1120	Sweet	3.0	0.5 ± 0.1 d	1.5 ± 0.1 b	1.5 ± 0.1 b	1.0 ± 0.1 c	0.5 ± 0.1 d	1.06 ± 0.00 b	1.09 ± 0.00 a	0.92 ± 0.00 c	0.43 ± 0.00 d	0.42 ± 0.00 d
Hexanal	1076	Green, grassy	1.2	1.0 ± 0.1 b	1.0 ± 0.1 b	1.0 ± 0.1 b	1.5 ± 0.1 a	1.0 ± 0.1 b	5.37 ± 0.00 b	3.18 ± 0.00 d	3.03 ± 0.00 e	4.82 ± 0.00 c	5.87 ± 0.00 a
*β*-Ionone	1478	Violet-like, raspberry, floral	0.007	2.0 ± 0.1 b	2.0 ± 0.2 b	2.0 ± 0.2 b	2.0 ± 0.2 b	2.0 ± 0.1 b	100	100	100	100	100
*β*-Myrcene	988	Peppery, spicy, balsam	1.5	2.0 ± 0.2 a	2.0 ± 0.2 a	2.0 ± 0.2 a	2.0 ± 0.2 a	2.0 ± 0.2 a	14.75 ± 0.44 a	4.39 ± 0.06 d	4.26 ± 0.01 e	4.78 ± 0.05 c	4.83 ± 0.09 b
Nonanal	1386	Sweet	1.0	1.5 ± 0.1 a	1.5 ± 0.1 a	1.5 ± 0.1 a	1.5 ± 0.1 a	1.5 ± 0.1 a	16.81 ± 0.00 a	15.91 ± 0.00 c	15.96 ± 0.00 b	15.12 ± 0.00 d	14.97 ± 0.00 e
*β*-Ocimene	1045	Green, burnt aroma	0.07	1.5 ± 0.1 b	1.5 ± 0.1 b	1.5 ± 0.1 b	2.0 ± 0.1 a	1.5 ± 0.1 b	58.18 ± 1.74 a	18.80 ± 0.01 b	17.06 ± 0.04 e	17.16 ± 0.17 d	17.31 ± 0.33 c
Linalool	1535	Sweet	6	1.5 ± 0.1 a	1.5 ± 0.1 a	1.5 ± 0.1 a	1.5 ± 0.1 a	1.5 ± 0.1 a	2.59 ± 0.08 a	1.22 ± 0.00 b	1.15 ± 0.00 c	0.66 ± 0.01 e	0.68 ± 0.01 d
Sulcatone	1331	Sweet, fruity	50	0.5 ± 0.1 c	0.5 ± 0.1 c	0.5 ± 0.1 c	1.0 ± 0.1 b	0.5 ± 0.1 c	0.03 ± 0.00 b	0.02 ± 0.00 c	0.02 ± 0.00 c	0.02 ± 0.00 c	0.12 ± 0.00 a
Terpinolene	1276	Grassy	12.0	1.0 ± 0.1 a	1.0 ± 0.1 a	1.0 ± 0.1 a	0.5 ± 0.1 b	1.0 ± 0.1 a	0.04 ± 0.00 a	0.04 ± 0.00 a	0.04 ± 0.00 a	0.02 ± 0.00 b	0.02 ± 0.00 b
Undecanal	1205	Sweet	3.0	0.5 ± 0.1 b	0.5 ± 0.1 b	0.5 ± 0.1 b	0.5 ± 0.1 b	0.5 ± 0.1 b	0.34 ± 0.00 a	0.29 ± 0.02 b	0.27 ± 0.00 c	0.27 ± 0.00 c	0.26 ± 0.00 c

^a^ RIs were calculated from published retention times of each compound using a matching score threshold of 780 for the mass spectra in the NIST 20 database. ^b^ As stated in http://www.thegoodscentscompany.com. ^c^ [35], ^d^ [16]. Columns labeled with ‘a’, ‘b’, ‘c’, ’d’ and ’e’ had significant differences (*p* < 0.05) from each other.

## Data Availability

The original contributions presented in the study are included in the article/Supplementary Materials; further inquiries can be directed to the corresponding authors.

## Supplementary Materials

The following supporting information can be downloaded at: https://www.mdpi.com/article/10.3390/foods15173067/s1, Figure S1: Partial least squares-discriminant analysis (PLS-DA) of non-targeted metabolomics data from B1–B5. Pairwise comparisons of differential volatile organic compounds across processing stages: (A) B1 *vs*. B2, (B) B2 *vs*. B3, (C) B3 *vs*. B4, (D) B4 *vs*. B5.
