# Supplementary material for: Effects of Fixation and Drying on the Physicochemical Quality and Aroma Profiles of Vine Tea (Nekemias grossedentata): Identification of Critical Processing Steps for Flavor Formation
Source: Foods. 2026 Aug 29;15(17):3067. doi: 10.3390/foods15173067 (PMC13565024; doi:10.3390/foods15173067)
Supplement: Supplementary file 1 [file foods-15-03067-s001.zip › foods-4448741-supplementary.pdf]

Supplementary material

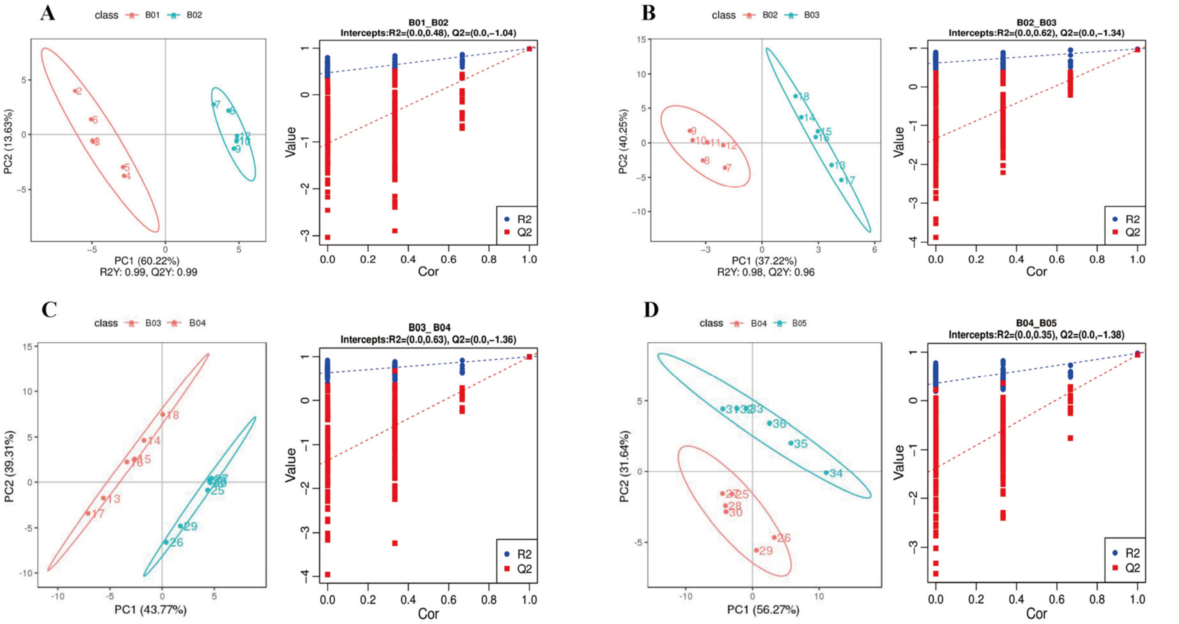

Figure S1. Partial least squares-discriminant analysis (PLS-DA) of non-targeted metabolomics data from B1–B5. Pairwise comparisons of differential volatile organic compounds across processing stages: (A) B1 *vs*. B2, (B) B2 *vs*. B3, (C) B3 *vs*. B4, (D) B4 *vs*. B5.
